# Temporal-Sequential Learning With a Brain-Inspired Spiking Neural Network and Its Application to Musical Memory

**DOI:** 10.3389/fncom.2020.00051

**Published:** 2020-07-02

**Authors:** Qian Liang, Yi Zeng, Bo Xu

**Affiliations:** ^1^Research Center for Brain-Inspired Intelligence, Institute of Automation, Chinese Academy of Sciences, Beijing, China; ^2^School of Artificial Intelligence, University of Chinese Academy of Sciences, Beijing, China; ^3^National Laboratory of Pattern Recognition, Institute of Automation, Chinese Academy of Sciences, Beijing, China; ^4^Center for Excellence in Brain Science and Intelligence Technology, Chinese Academy of Sciences, Shanghai, China

**Keywords:** spiking neural network, sequential memory, episodic memory, spike-timing-dependent plasticity, time perception, musical learning

## Abstract

Sequence learning is a fundamental cognitive function of the brain. However, the ways in which sequential information is represented and memorized are not dealt with satisfactorily by existing models. To overcome this deficiency, this paper introduces a spiking neural network based on psychological and neurobiological findings at multiple scales. Compared with existing methods, our model has four novel features: (1) It contains several collaborative subnetworks similar to those in brain regions with different cognitive functions. The individual building blocks of the simulated areas are neural functional minicolumns composed of biologically plausible neurons. Both excitatory and inhibitory connections between neurons are modulated dynamically using a spike-timing-dependent plasticity learning rule. (2) Inspired by the mechanisms of the brain's cortical-striatal loop, a dependent timing module is constructed to encode temporal information, which is essential in sequence learning but has not been processed well by traditional algorithms. (3) Goal-based and episodic retrievals can be achieved at different time scales. (4) Musical memory is used as an application to validate the model. Experiments show that the model can store a huge amount of data on melodies and recall them with high accuracy. In addition, it can remember the entirety of a melody given only an episode or the melody played at different paces.

## 1. Introduction

The human brain is a powerful machine for processing information about the world as it changes over time. Much of the knowledge that is acquired by a person in daily life is stored and retrieved in ordered sequences of, for example, actions, sounds, and images. Episodic memory allows a person to relive an event from their recollections of that event in space and time, in what can be termed the context of sequential events. Thus, remembering the order of information is critical for decision-making, prediction, planning, and other cognitive behaviors. It is evident that sequential memory is a fundamental part of the memory system. However, it is an extremely complex process.

Over the past few decades, a variety of methods based on different theories have been developed to model sequential memory processes, and attempts have been made to apply these methods to practical problems. In particular, one traditional machine learning technique, recurrent neural networks (RNNs) (Hochreiter and Schmidhuber, 1997; Schuster and Paliwal, 1997), has been widely used to process sequential learning tasks, such as natural language processing (Elman, 1990; Socher et al., 2010; Sutskever et al., 2014), video classification and representation (Srivastava et al., 2015; Yue-Hei Ng et al., 2015), speech enhancement and recognition (Graves et al., 2013; Weninger et al., 2015), human action recognition (Du et al., 2015; Liu et al., 2016), and musical learning (Eck and Schmidhuber, 2002; Eck and Lapalme, 2008). Although they have been studied in depth and used widely, RNNs are incapable of operating as efficiently as the brain. It has been shown that these models have limited capacity for long-term storage of information. Their ability to represent sequential data also appears to be limited. Furthermore, tuning of the parameters is a time-consuming and very difficult task, since the computational processes, and indeed the whole network, on which these models are based have no clear biological interpretations. These drawbacks have motivated the development of various other models inspired by the brain. A supervised learning model called sequential-temporal memory (Hu et al., 2016) aims to learn ordered information based on the formation of neural assemblies. This model has been adapted to a hardware architecture (Liu et al., 2019) to analyze associative memory and episodic memory. Another spike-based model (Tully et al., 2016) learns and recalls sequences in combination with Bayesian theory. George and Hawkins (2009) and Hawkins and Ahmad (2016) have presented a hierarchical temporal memory network inspired by cortical structure to encode and learn sequential features. This model has been used, for example, for prediction and anomaly detection. A model that is capable of learning and reproducing sequences based on functional networks and which has the potential for memory and rhythm generation has been described by Verduzco-Flores et al. (2012).

In this paper, we develop a spike-based model that is inspired by the human brain and is capable of storing and retrieving a large number of musical pieces. To provide a basis for the work, we first need to understand the mechanisms of sequential memory as well as the areas of the brain that are involved and the ways in which they cooperate. There have been many investigations into how the brain learns and preserves sequential events. It has been found that the hippocampus plays a critical role in encoding and preserving temporal orders of sequences (Davachi and Dubrow, 2015). In some species, the activity of “place cells” occurs in the same order as prior experience (Skaggs et al., 1996). “Time cells” may encode successive moments between events, temporal location, and even ongoing behavior (MacDonald et al., 2011). As well as the medial temporal lobe (MTL), the prefrontal cortex (PFC) and the striatum also contribute to temporal memory (McAndrews and Milner, 1991; Tubridy and Davachi, 2011; Meier et al., 2013). One study reported that the activities of the MTL and PFC are enhanced when information is represented and retrieved during the establishment of temporal context memory (Jenkins and Ranganath, 2010). It has been found that the sensory cortex (auditory, visual, and motor cortex) also has a role in sequential memory. Taking these findings together, this paper attempts to bridge the gap between traditional models and the real brain. We construct a neural network model inspired by relevant evidence and validate the model using musical examples. Compared with existing models, the innovative aspects of this work are as follows:

- The model is to some extent biologically plausible. It is composed of several collaborative subnetworks that are similar to corresponding areas of the brain. Three critical processes—encoding, storage, and retrieval—are involved in sequential memory and episodic memory.
- A dependent timing module is modeled according to the mechanisms of time perception in the brain. Time intervals between sequential elements are perceived by temporal minicolumns, and a pacemaker population is introduced to control the speed of the retrieval process.
- For individual neurons, the Izhikevich model is adopted and can simulate multiple spiking patterns of neurons.
- Synaptic connections (including two types, excitatory and inhibitory) with different transmission delays are included in the model and exist between neurons from any layers. Contextual memory can be represented with connections from different layers. A spike-timing-dependent plasticity learning rule allows the network to modulate weights during the learning process.
- The numbers of neurons and synapses change dynamically during the learning process, which makes the model more flexible.
- Musical memory is a typical example of sequential memory. We use the model to encode and store many melodies and retrieve them based on a MIDI dataset (Krueger, 2018).

The remainder of the paper has the following structure: section 2 describes the model and the associated methods. Section 3 presents the experimental results. Section 4 gives a summary and discusses possible future work.

## 2. Model and Methods

### 2.1. Model Description

The model is composed of four neural clusters. The functions of these clusters are similar to those of specific brain areas. It should be noted that our goal is to design an efficient network rather than merely simulate the brain.

#### 2.1.1. Network Architecture

**Figure 1:**
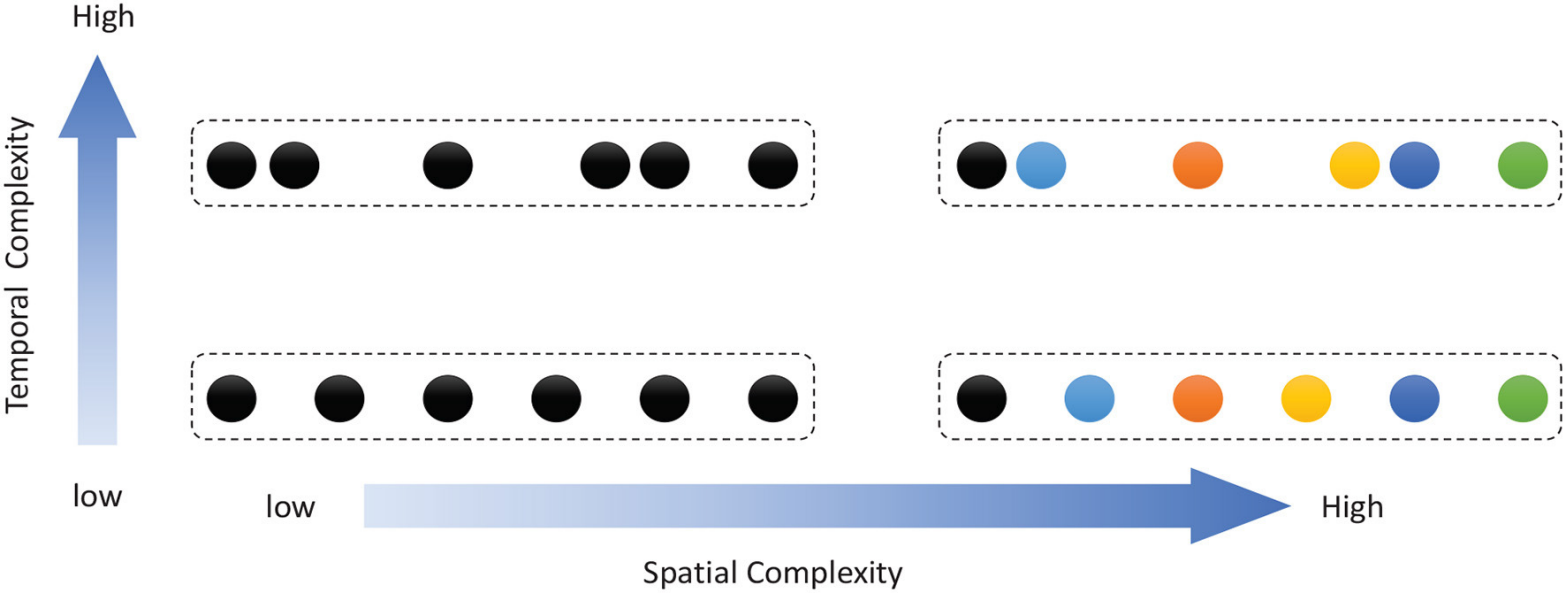
Description of sequential spatial and temporal patterns: the color of a circle represents the content of a sequential element, and the distance between two circles represents the time interval.

**Figure 2:**
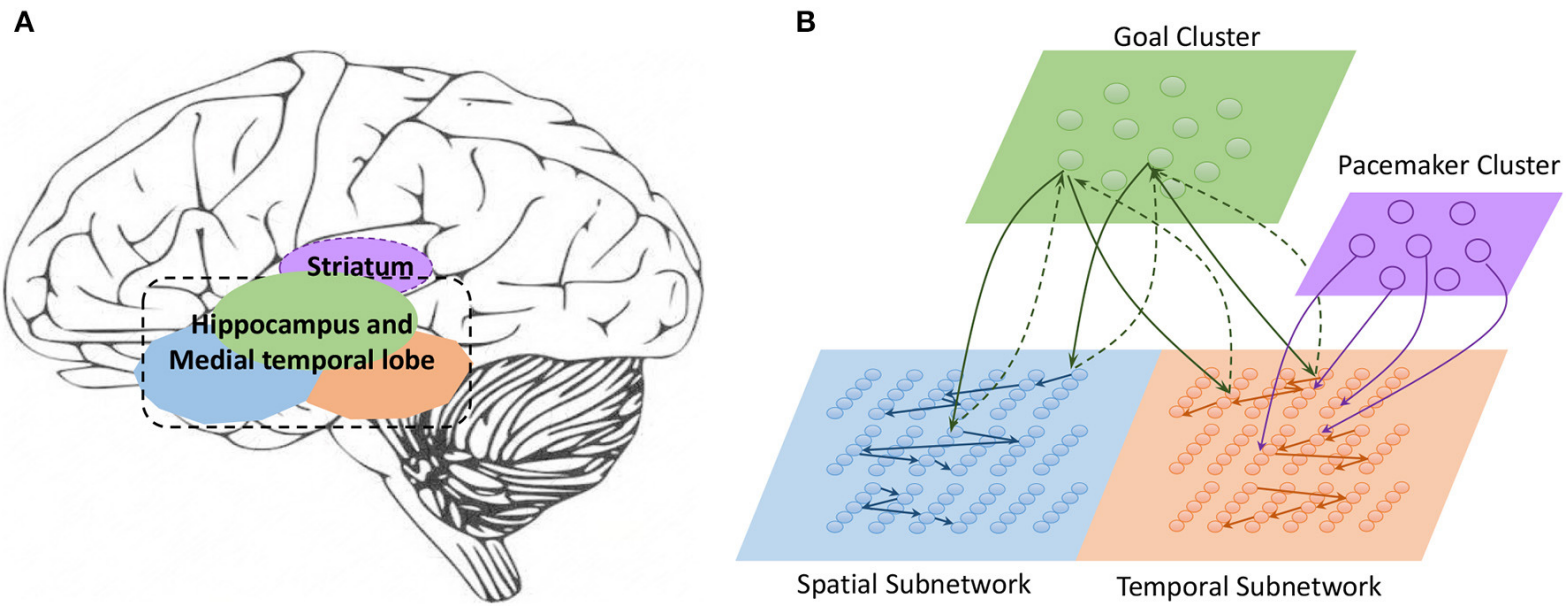
**(A)** Main regions of the brain related to sequential memory. **(B)** Architecture of the model: the green area is the goal cluster, the purple area is the pacemaker cluster, and the blue and orange areas are the spatial and temporal subnetworks, respectively; these areas collaborate via their connections.

Work on rodents has indicated that hippocampal place cells encode ordered sequences or positions and may predict upcoming locations (Lisman and Redish, 2009). Time cells in the MTL fire at successive moments in ordered, structured events and work in parallel with place cells (Eichenbaum, 2014). Furthermore, the results of recent studies have highlighted the role of the hippocampus in representation and retrieval in episodic memory (Fortin et al., 2002). Meanwhile, it has been shown that cortical-basal ganglia loops play an important role in time perception (Buhusi and Meck, 2005; Merchant et al., 2013a), and it has been found that striatal populations, in particular, can encode relative time and adjust animal behaviors (Matell and Meck, 2004; Mello et al., 2015). Inspired by all of these results, we design the underlying architecture of the model. Before describing this in detail, we first define two features of a sequence, namely, the spatial pattern and temporal pattern, as shown in Figure 1. A collection of the contents of sequential elements can be considered a spatial pattern, and a collection of time intervals between these elements can be considered a temporal pattern. Our network is an associative model and contains four interrelated subnetworks inspired by different brain regions (Figure 2A). The relationships between the subnetworks are shown in Figure 2B.

**Figure 3:**
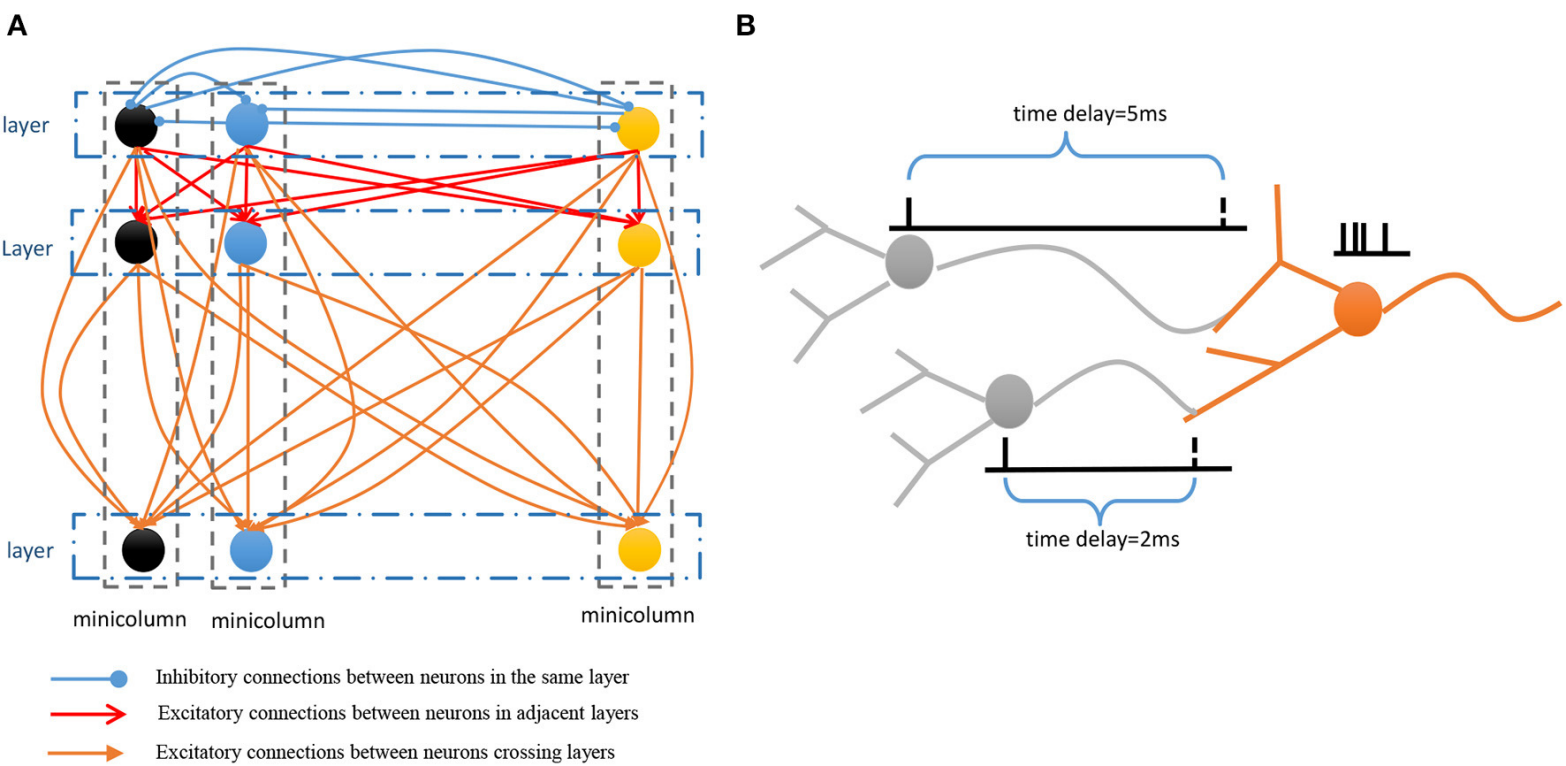
**(A)** Connection architecture of spatial and temporal subnetworks. **(B)** Synaptic connections have different transmission delays between neurons.

- **Spatial subnetwork**. The blue area is the spatial subnetwork, which mainly encodes spatial patterns and learns the order of sequential elements. This subnetwork consists of a series of non-overlapping neural minicolumns, and its connections are shown in Figure 3A. A minicolumn is composed of about 100 neurons and has a small but specific function. Each organization of horizontal neurons is called a layer. Connections between neurons in the same layer are inhibitory, with only one neuron being excited at a given time. Connections from adjacent layers are excitatory and represent the ordered information of sequential elements, and connections between neurons that cross layers carry history information. To improve the network performance, a mechanism for transmission delay is introduced (Swadlow, 1985, 1988, 1992), by which the transmission delay of a connection is set proportional to the number of layers between the neurons (see Figure 3B). This means that action potentials have long journeys on long connections. However, the transmission delays are restricted to lie in the range 0–60 ms, so action potentials decay to 0 mV after 60 ms, and postsynaptic neurons cannot receive spikes that travel for more than 60 ms along connections. The experimental results show that these connections are crucial for context memory.
- **Temporal subnetwork**. The orange area in Figure 2B is the temporal subnetwork, which is responsible for representing and storing temporal information between sequential elements. Just like the spatial subnetwork, the temporal subnetwork is composed of minicolumns in which neurons are sensitive to the length of the time interval. The connection architecture of this subnetwork is the same as that of the spatial subnetwork.
- **Goal cluster**. The green area in Figure 2B represents the “goal” or “label” cluster to which a sequence belongs; for example, these clusters could be names of songs or goals of actions. It is reasonable to assume that a goal or a label can be activated at the same time during learning and retrieval of a series of events. This area contains numerous neurons that represent different goals of sequences associated with the subnetworks processing spatial and temporal patterns. In contrast to the spatial and temporal subnetworks, there are no internal connections between neurons in this cluster. Moreover, external synaptic connections between the “goals” cluster and the other two subnetworks are dynamically generated during the learning process.
- **Pacemaker cluster**. The purple area in Figure 2B is called the pacemaker cluster, which works like a pacemaker to adjust time scales during the retrieval process. All neurons in this cluster project their feedforward connections to neurons in the temporal subnetwork. This cluster also lacks internal connections. The mean firing rate of this population controls the speed of the retrieval process. For example, a person can play the same melody on a piano at different speeds.

#### 2.1.2. Neural Dynamics

Individual neuronal dynamics are described using the Izhikevich spiking model (Izhikevich, 2003). This model is a two-dimensional non-linear model and is more computationally efficient than the Hodgkin-Huxley model (Hodgkin and Huxley, 1952). The Izhikevich neuronal model can be expressed in terms of the two equations

(1) $$\begin{array}{l} \frac{dv}{dt}=0.04v^{2}+5v+140-u+I, \end{array}$$

(2) $$\begin{array}{l} \frac{du}{dt}=a\left(bv-u\right). \end{array}$$

The variables *u* and *v* are reset according to the following conditions after emission of a spike:

(3) $$\begin{array}{l} \text{if}v\geq 30\text{mV, then}\{\begin{array}{l} v\leftarrow c,\\ u\leftarrow u+d. \end{array} \end{array}$$

Here, *v* represents the membrane potential of a neuron, and *u* is a membrane recovery variable; *a*, *b*, *c*, and *d* are parameters that modulate the model to adapt to different spiking patterns. The *I* in Equation (1) is the input current, which carries information from external stimuli and from other neurons. When the membrane potential reaches the peak value (30 mV), the neuron emits a spike and *u* and *v* are reset, after which the neuron will be silent for a while. Here, we use the regular spiking pattern described by Izhikevich (2003), with the parameters *a* = 0.02, *b* = 0.2, *c* = −65, and *d* = 8.

#### 2.1.3. Synaptic Plasticity

Synaptic plasticity is a biological mechanism that adjusts the strength of neuronal connections during the learning process. This paper uses spike-timing-dependent plasticity (STDP) (Bi and Poo, 1998) to modulate network connections. According to this learning rule, if a presynaptic neuron fires just before the postsynaptic neuron within a short time window, then the synaptic strength will be increased; otherwise, it will decrease. These two forms of change in synaptic strength are called long-term potentiation and long-term depression, respectively. The STDP learning rule can be described as follows in terms of the total synaptic weight change *W*_syn_ induced by stimulation by *N* pairs of presynaptic and postsynaptic spikes:

(4) $$\begin{array}{l} W_{\text{syn}}\left(i,j\right)=\overset{N}{\underset{f=1}{\sum }}\overset{N}{\underset{n=1}{\sum }}\Delta w\left(t_{i}^{n}-t_{j}^{f}\right), \end{array}$$

where $t_{j}^{f}$ is the arrival time of presynaptic spike *f* at synapse *j* and $t_{i}^{n}$ is the firing time of the *n*th spike at postsynaptic neuron *i*. The STDP function (or learning window) is given by

(5) $$\begin{array}{l} \Delta w\left(x\right)=\{\begin{array}{cc} A_{+}e^{-x/\tau_{+}}, & x>0,\\ -A_{-}e^{x/\tau_{-}}, & x<0, \end{array} \end{array}$$

where *A*_+_ and *A*_−_ are parameters for adjusting the weights, τ_+_ and τ_−_ are time constants, and *x* = *t*_*i*_ − *t*_*j*_ denotes the time difference between the presynaptic and postsynaptic spikes.

### 2.2. Encoding

Encoding information is an extremely important but difficult task. Population coding (Hu et al., 2016) and sparse coding (Byrnes et al., 2011) have been used to encode sequences. In fact, neurons within a given region in the nervous system always have identical receptive fields and also encode similar features. For example, regions of the cochlear nucleus, located in the subcortical part of the auditory pathway, are stimulated selectively by different sound frequencies and exhibit sustained spiking activity (Mcdermott and Oxenham, 2008; Oxenham, 2012). Orientation minicolumns located in the primary visual cortex of cats and other mammals respond to their preferred directions (Hubel and Wiesel, 1959, 1968). In macaque motor areas, cells organized into a group prefer specific direction vectors (Amirikian and Georgopoulos, 2003). Evidence has also been found that modules of cells in the “rewired” auditory cortex share a preferred orientation during the receipt of inputs from the retina (Sharma et al., 2000). There are a large number of minicolumns distributed widely in the cortex that implement various functions. In this paper, both spatial and temporal subnetworks are constructed using functional minicolumns as building blocks. The main ideas of the encoding process are that (1) neurons located in the same minicolumn have the same preference, (2) the Izhikevich neural model is used to simulate the neurons and transform preferred information into spike activities, (3) the input current of the Izhikevich neural model, denoted by *I* in Equation (1), is computed by a Gaussian filter.

#### 2.2.1. Encoding of Spatial Patterns

A sequence can be defined as a collection

$$S=\left\{x_{1},t_{12},x_{2},t_{23},x_{3},\ldots ,x_{r},t_{rr+1},x_{r+1},\ldots ,x_{n-1},t_{n-1n},x_{n}\right\}$$

where *x*_*r*_ denotes the content of a sequential element and *t*_*rr*+1_ denotes the time interval between *x*_*r*_ and *x*_*r*+1_. Then, a sequence can be divided into two sets, SP = {*x*_*r*_ ∣ *r* = 1, 2, …, *n*} and TP = {*t*_*rr*+1_ ∣ *r* = 1, 2, …, *n* − 1}, which represent spatial and temporal patterns, respectively.

**Figure 4:**
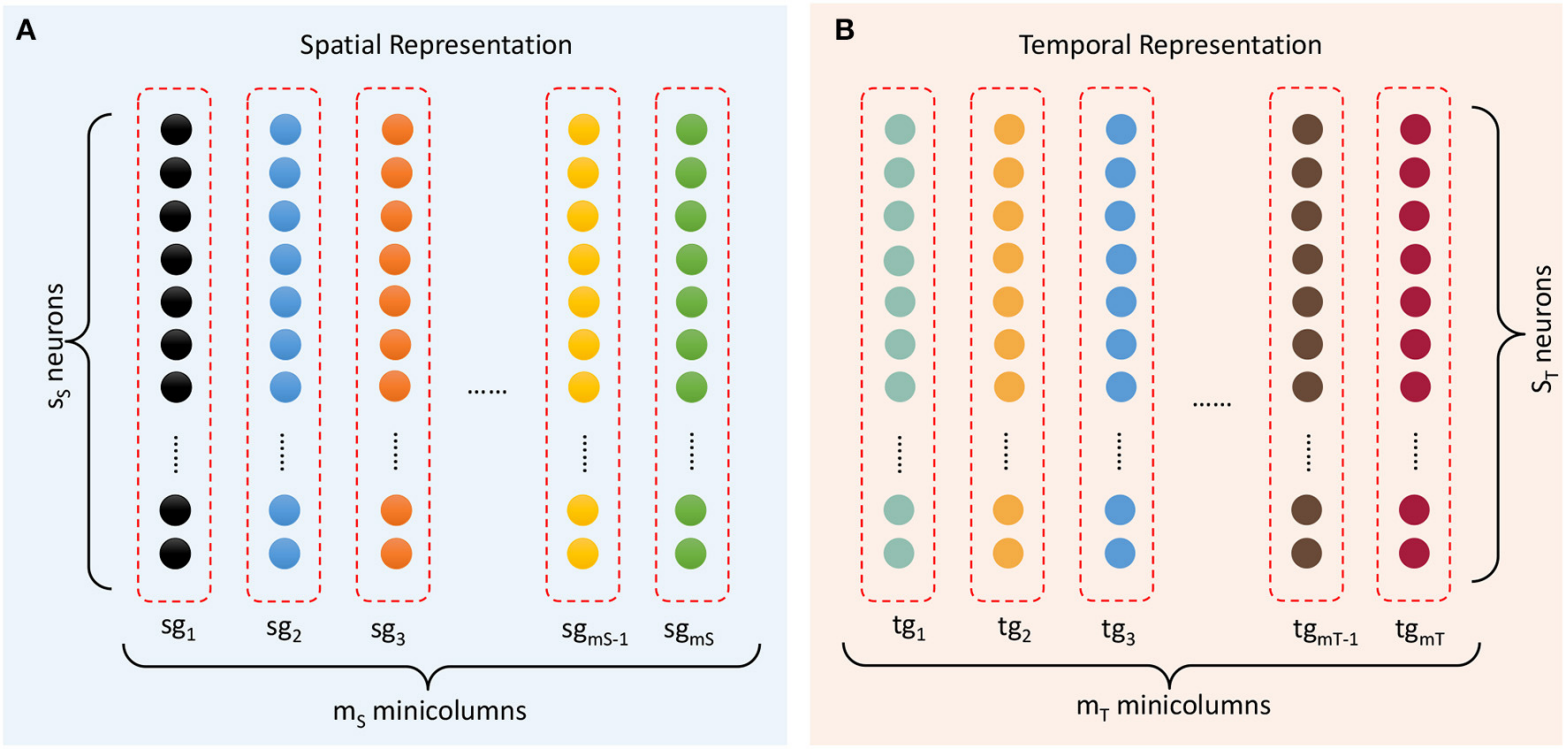
Representation of spatial and temporal patterns. **(A)** Minicolumns that prefer the contents of sequential elements. **(B)** Temporal minicolumns, which are sensitive to time intervals.

The spatial subnetwork contains *m*_*S*_ minicolumns (as shown in Figure 4A) and can be defined as {*sg*_*i*_ ∣ *i* = 1, …, *m*_*S*_}. Each vertical group *sg*_*i*_ is considered a minicolumn in which neurons SN_*i*_ = {*sn*_*ij*_ ∣ *j* = 1, …, *s*_*S*_} have identical selectivity (marked in the same color) for the same content. The selectivity of a neuron can be interpreted as a preference or a filter of external stimulation. This property transforms an external stimulus into a current. In reality, neurons with a specific selectivity can be triggered within a range around a preferred input rather than at a precise value. Hence, the current resulting from external stimulation of each neuron *sn*_*i,j*_ is computed by a Gaussian filter as follows:

(6) $$\begin{array}{l} I_{s\text{\_}{\text{ext}}_{ij}}=k_{1}\frac{1}{\sqrt{2\pi }\sigma_{si}}e^{-{\left(x_{r}-\mu_{si}\right)}^{2}/\sigma_{si}^{2}}, \end{array}$$

where *x*_*r*_ is the external stimulus, and μ_*si*_ and σ_*si*_ are respectively the mean and variance of the preference of the neuron *sn*_*ij*_. This current is then used as the input *I* to the Izhikevich neural model; see Equation (1). Neurons in the same minicolumn *sg*_*i*_ have the same μ_*si*_ and σ_*si*_, and *k*_1_ is a modulating coefficient to make the current strength suitable for the Izhikevich model. Actually, μ_*si*_ and σ_*si*_ can be interpreted as the neural preference and the range of preference, respectively. The closer *x*_*r*_ is to the preference μ_*si*_, the larger *I*_*s*_ext_*ij*__ is. Since *I*_*s*_ext_*ij*__ is the input current of the Izhikevich model, the neuron exhibits sustained spike activity if *I*_*s*_ext_*ij*__ is large enough. In other words, this formula means that neurons will emit spikes if they prefer *x*_*r*_; otherwise, they will be resting. Usually, the range of the neural receptive field is small. Therefore, σ_*si*_ is set to a correspondingly small value.

#### 2.2.2. Encoding of Temporal Patterns

Time perception is another critical issue in this study. At present, there is no consensus among researchers in this field. Merchant and his team (Merchant et al., 2013a) summarized three possible timing mechanisms. One theory is that the basal ganglia-cerebellum-thalamus is the common timing system, whereas according to another theory timing is an intrinsic capability of any cortical circuit (Gupta, 2014). A third theory postulates that both of the preceding mechanisms are present in the brain and that they interact with each other. In this paper, we adopt the second theory to encode time intervals. It has been found that a large population of medial premotor cortex cells are tuned to various signal durations, with a distribution of preferred durations covering all intervals in hundreds of milliseconds (Merchant et al., 2013a; Gupta, 2014). Cells found in other sensory cortices have similar properties (Merchant et al., 2013a,b). Based on these mechanisms, encoding of temporal patterns can be achieved in a similar way to that of spatial patterns.

The temporal subnetwork {*tg*_*i*_ ∣ *i* = 1, …, *m*_*T*_} is shown in Figure 4B. Neurons TN_*i*_ = {*tn*_*ij*_ ∣ *j* = 1, …, *s*_*T*_} in minicolumn *tg*_*i*_ all have the same preferred duration. Each neuron receives the input time interval *t*_*rr*+1_ and generates a current

(7) $$\begin{array}{l} I_{t\text{\_}{\text{ext}}_{ij}}=k_{2}\frac{1}{\sqrt{2\pi }\sigma_{ti}}e^{-{\left(x_{rr+1}-\mu_{ti}\right)}^{2}/\sigma_{ti}^{2}} \end{array}$$

where *x*_*rr*+1_ is the time interval between two spatial elements. The mean μ_*ti*_ and variance σ_*ti*_ of neurons in minicolumn *tg*_*i*_ are set to adapt the scope of their preferred time interval; *k*_2_ is also an experiential value. The neuronal dynamics are then computed using Equation (1). In this way, time intervals are transformed into corresponding neuronal activities. It is important to note that, based on the time perception mechanisms mentioned above, our model expands the perceptual scope of durations from tens of milliseconds to a few seconds to satisfy the demands of practical applications.

#### 2.2.3. Encoding of the Goal Cluster

**Figure 5:**
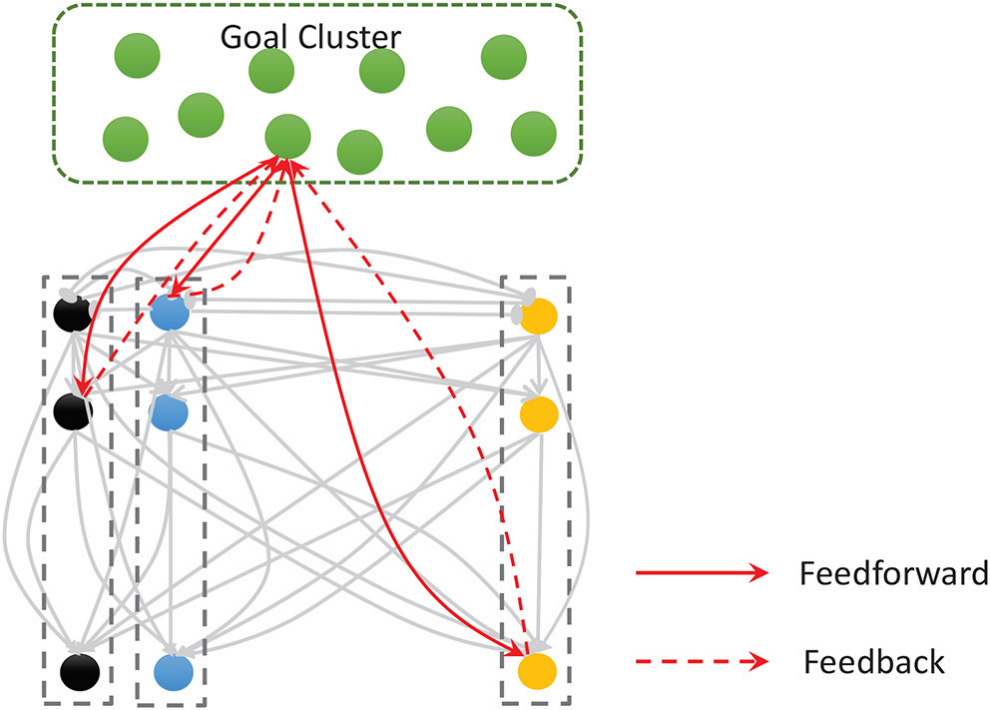
The goal cluster sends feedforward and feedback connections to the spatial and temporal subnetworks. The internal connections of these two subnetworks are shown here as gray arrows.

The goal cluster contains numerous neurons rather than minicolumns, as shown in Figure 5. Each neuron stands for the label (goal) of a sequence; in other words, a label (goal) is set as a neural preference.

During the encoding process, the external stimulation is a label (e.g., the name of a musical piece), and all the neurons in this cluster are traversed to find the one whose preference matches the external stimulation. If the match is successful, we inject a 20 mA current into the neuron directly, rather than using a Gaussian filter. Neurons in this cluster are also simulated using the Izhikevich regular spiking neuronal model.

### 2.3. Storage

Sequence storage is an associative process in which spatial patterns, temporal patterns, and goals are concentrated into network circuits simultaneously. Storage is based on the encoding process: ordered sequential elements lead to neurons firing in an orderly manner, and thereby connections between these neurons are potentiated or reduced by the STDP learning rule. At the beginning of this process, the model is empty. Then, the model learns sequential elements one by one. To explain the model clearly, we use a sample sequence, written as *G*_1_:{*B*, 270 ms, *A*, 330 ms, *D*, 230 ms, *C*} where *G*_1_ denotes the goal, to describe the storage process.

**Figure 6:**
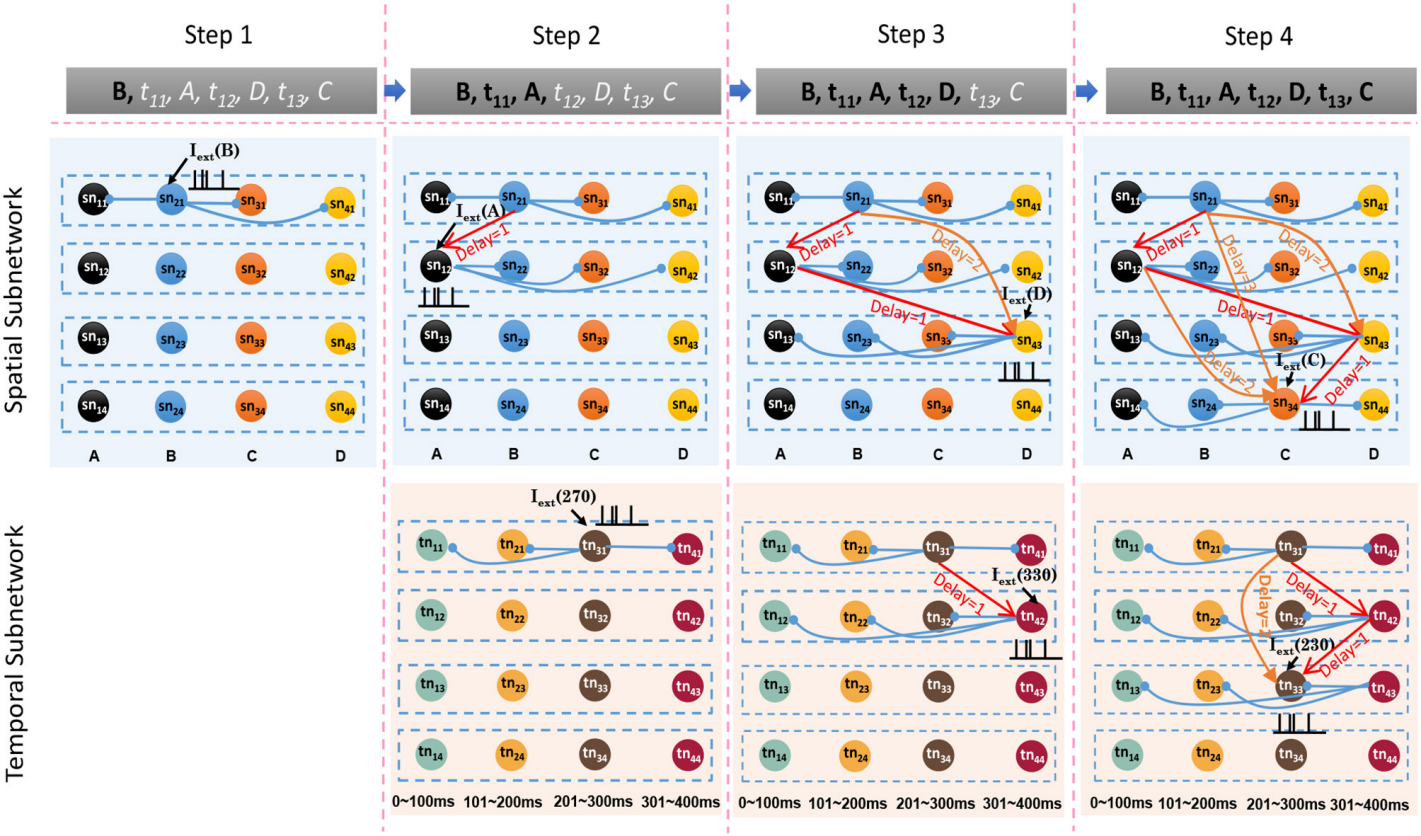
Synaptic connections in the spatial and temporal subnetworks are trained during the learning process.

The spatial and temporal patterns of the sample are SP(*G*_1_) = {*B, A, D, C*} and TP(*G*_1_) = {270 ms, 330 ms, 230 ms}. Figure 6 shows the learning process of the spatial and temporal subnetworks with the input of the sample.

- **Step 1**. Sequential element *B* triggers the minicolumn preferring *B* (marked in blue), and neuron *sn*_21_ responds to this stimulation. This neuron then fires and inhibits other neurons in the same layer. The time duration for which each neuron continues to fire is set to 3 ms, after which the membrane potential decays to 0 mV.
- **Step 2**. After 270 ms, element *A* triggers the firing of a neuron *sn*_12_ in the minicolumn that prefers *A*. Simultaneously neuron *tn*_31_, which represents a time interval of 270 ms between *B* and *A* in the temporal subnetwork, fires. Because of internal conduction delay in the spatial subnetwork, this neuron exactly receives the spikes of neuron *sn*_21_, and the synaptic weight between these two firing neurons is enhanced by the STDP learning rule. It is important to note that conduction delays only help connections to store the order between neurons, while real-time intervals are stored by temporal neurons.
- **Step 3**. Element *D* leads to the firing of neuron *sn*_43_, and the connection representing the ordered information between this neuron and *sn*_12_ is enhanced. The contextual connection between *sn*_21_ and *sn*_43_ (marked by the orange arrow) is strengthened owing to the exact arrival of spikes. This means that historical contexts have an impact on current neuronal activities. Meanwhile, neuron *tn*_42_ emits spikes because it is sensitive to a time interval of 330 ms. The connection between *tn*_31_ and *tn*_42_ is also strengthened.
- **Step 4**. The last element *C* and the time interval 230 ms excite the neurons *sn*_34_ and *tn*_33_ in the spatial and temporal networks, respectively. Contextual connections with different time delays are also updated.

**Figure 7:**
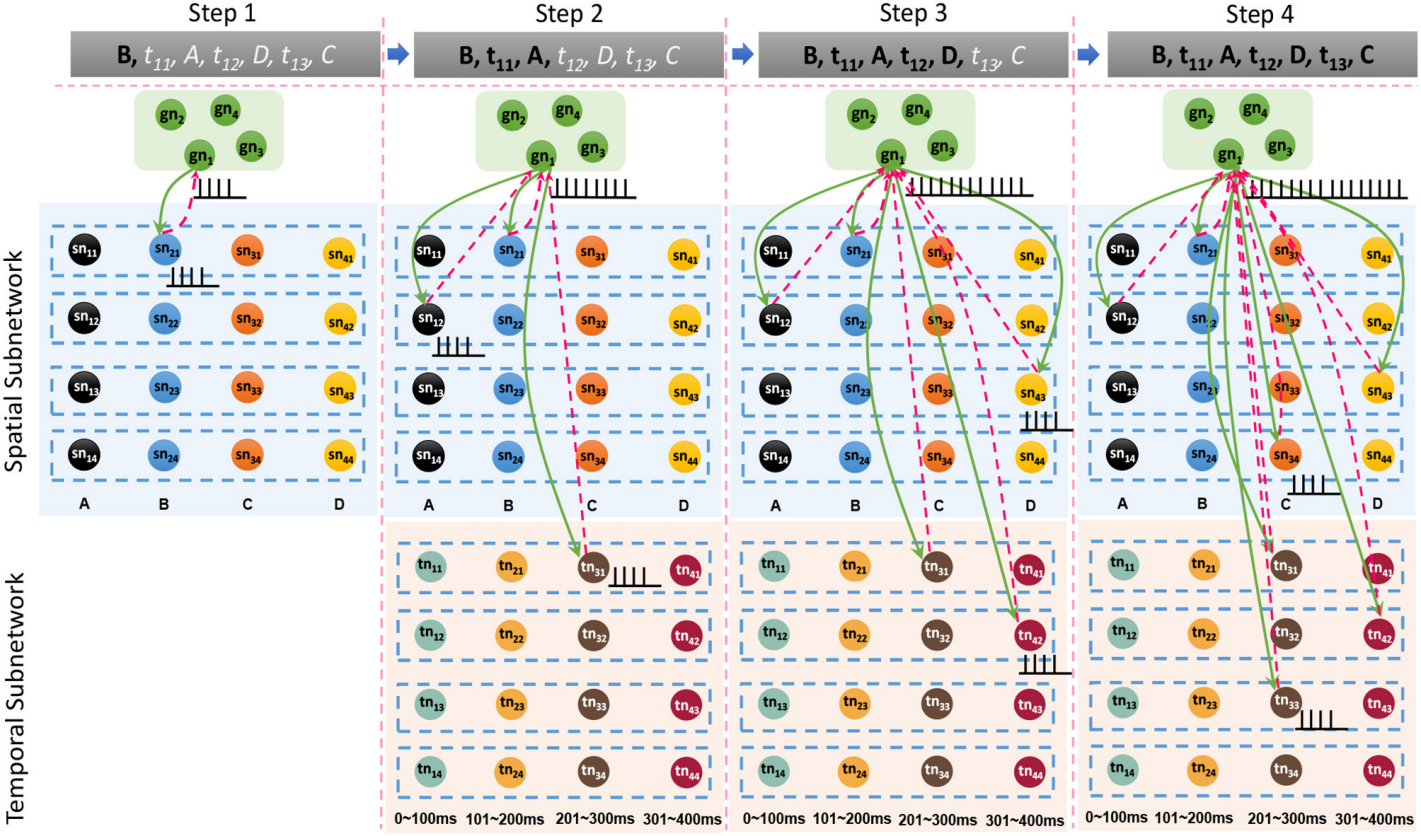
Synaptic connections between the goal cluster and the spatial and temporal subnetworks are dynamically generated and trained during the storage process.

Besides the internal learning processes of the spatial and temporal subnetworks, external connections between the goal cluster and these two subnetworks are generated and updated simultaneously. A neuron allocated to encode *G*_1_ and labeled as *gn*_1_ in the goal cluster fires continuously until the end of the last learning step. In fact, *gn*_1_ and other firing neurons in the spatial and temporal subnetworks form resonant relationships because of their similar neuronal spiking patterns. As shown in Figure 7, in each step, synaptic (including feedforward and feedback) connections between *gn*_1_ and neurons in the spatial subnetwork are generated first because of their synchronous oscillations, after which feedforward connections (green arrows) are updated by the STDP learning rule. Connections between *gn*_1_ and neurons in the temporal subnetwork are also generated and updated in the same way. However, the weights of the feedback connections (red dashed arrows) are set to fixed values; this is designed to reduce the computational cost, but has yet to be supported by neurobiological findings.

Overall, we can conclude that neurons in the spatial and temporal subnetworks receive multiple types of currents. Therefore, the input current *I* of each neuron can be computed as

(8) $$\begin{array}{l} I\left(i\right)=w_{s}I_{\text{same}}+w_{a}I_{\text{adj}}+w_{c}I_{\text{crossing}}+w_{g}I_{\text{goal}}, \end{array}$$

where *I*(*i*) denotes the input current of any neuron *i* in either the spatial or the temporal subnetwork, since computational processes are the same in these subnetworks, *I*_same_ is the input current from neurons in the same layer, *I*_adj_ is the current from neurons in the adjacent layer, *I*_crossing_ is the current from neurons crossing layers, and *I*_goal_ is the current from the neuron of the goal cluster; *w*_*s*_, *w*_*a*_, *w*_*c*_, and *w*_*g*_ are empirical weights of these respective currents. The four types of currents are computed as follows:

(9) $$\begin{array}{l} I_{\text{same}}\left(i\right)=\underset{j\in n_{\text{same}}}{\sum }W_{\text{syn}}\left(i,j\right), \end{array}$$

(10) $$\begin{array}{l} I_{\text{adj}}\left(i\right)=\underset{j\in n_{\text{adj}}}{\sum }W_{\text{syn}}\left(i,j\right), \end{array}$$

(11) $$\begin{array}{l} I_{\text{crossing}}\left(i\right)=\underset{j\in n_{\text{crossing}}}{\sum }W_{\text{syn}}\left(i,j\right), \end{array}$$

(12) $$\begin{array}{l} I_{\text{goal}}\left(i\right)=\underset{j\in n_{\text{goal}}}{\sum }W_{\text{syn}}\left(i,j\right), \end{array}$$

where *n*_same_, *n*_adj_, *n*_crossing_, and *n*_goal_ denote collections of neurons from the same layer, from the adjacent layer, crossing layers, and from the goal cluster, respectively, and *W*_syn_(*i, j*) is the current between presynaptic neuron *n*_*i*_ and postsynaptic neuron *n*_*j*_ computed from the STDP learning rule in Equation (4).

### 2.4. Retrieval

We focus mainly on two types of sequential memory retrieval. One is goal-based retrieval, in which a whole sequence, including spatial and temporal patterns, is recalled given only goal information; for example, given the name of a melody, the musical sequence can be remembered. In the other type, called contextual retrieval, the associative sequential elements and the goal are recalled gradually, given only contextual information.

#### 2.4.1. Goal-Based Retrieval

**Figure 8:**
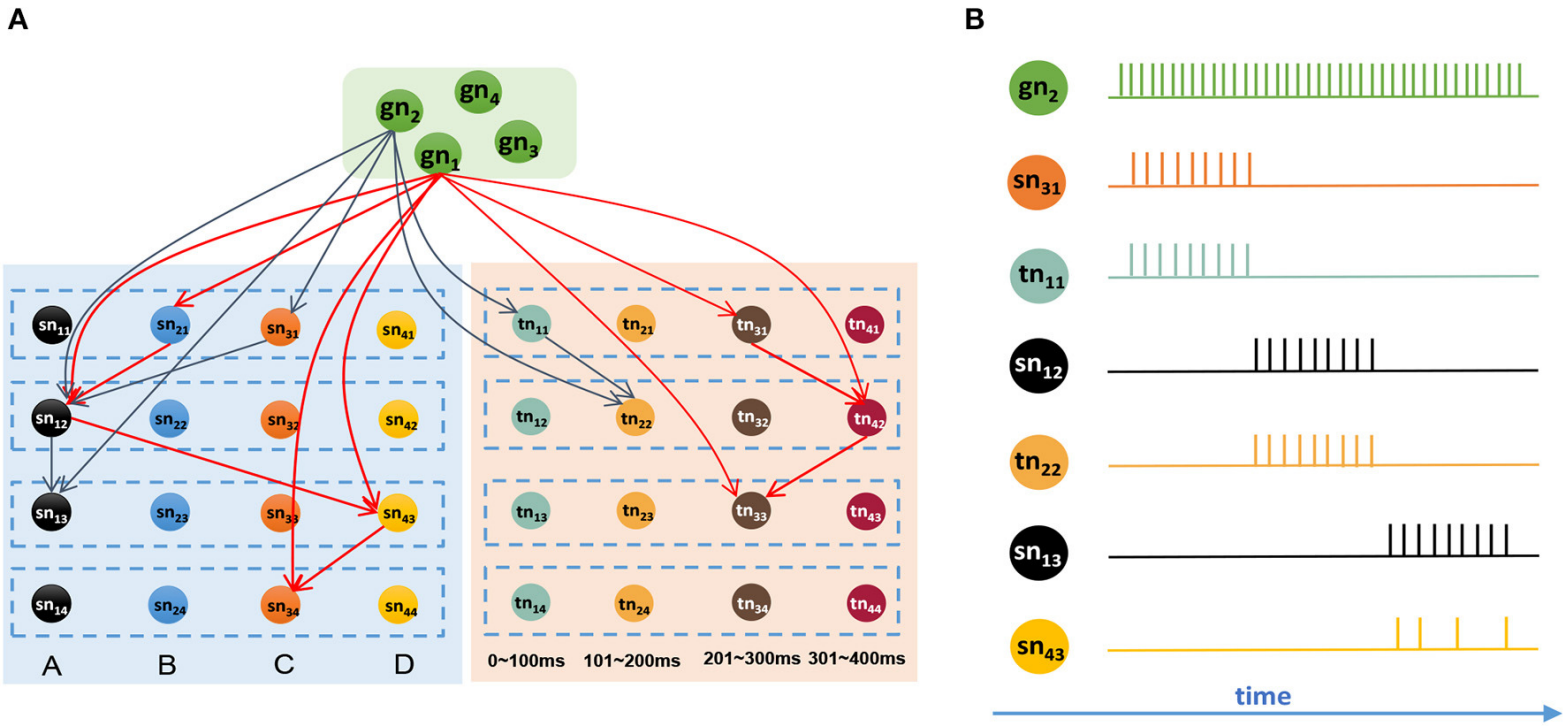
Goal-based retrieval: ordered activities of neurons in different clusters. **(A)** Synapses between the goal cluster and the spatial and temporal subnetworks; there are two sequences that have been stored in the model, and synapses with the same color belong to the same sequence. **(B)** Activities of neurons during the retrieval process.

It is reasonable to remember all the sequential events after giving the goal information. Figure 8A shows a network that has memorized two sequences, *G*_1_ = {*B*, 270 ms, *A*, 330 ms, *D*, 230 ms, *C*} and *G*_2_ = {*C*, 60 ms, *A*, 180 ms, *A*}. Connections in this graph are simplified, and only adjacent and goal-connected synapses are shown.

Suppose we are going to recall *G*_2_ represented by *gn*_2_. In this case, neuron *gn*_2_ will receive a strong external stimulus and will emit abundant spikes. Because of the trained synapses, *sn*_31_ and *tn*_11_, which encode *C* and 60 ms respectively, will be triggered and emit spikes first. Then, neurons *sn*_12_ and *tn*_22_ will receive the currents from goal information *gn*_2_ as well as contextual signals from *sn*_31_ and *tn*_11_, respectively, and will rapidly release action potentials. After *sn*_12_ fires, neurons *sn*_13_ and *sn*_43_ will receive signals because of the trained synapses. Since *sn*_13_ receives currents not only from *sn*_12_ but also from *gn*_2_, it will release spikes first, sending inhibitory signals to *sn*_43_ on account of the inhibitory synapse between them, and will exhibit the maximum firing rate. Here, we use the winner-take-all principle, and *sn*_43_ will decay gradually and eventually fail in this competition. Figure 8B shows the spiking sequences over time during this process.

#### 2.4.2. Contextual Retrieval

Humans have the capability of episodic memory, which includes not only the content of an event but also temporal information. It has been found that the hippocampus, MTL, and PFC are heavily involved in the contextual retrieval process (Jenkins and Ranganath, 2010). Inspired by these findings, our spiking neural network can also implement this important memory process. We present an example to explain the process.

**Figure 9:**
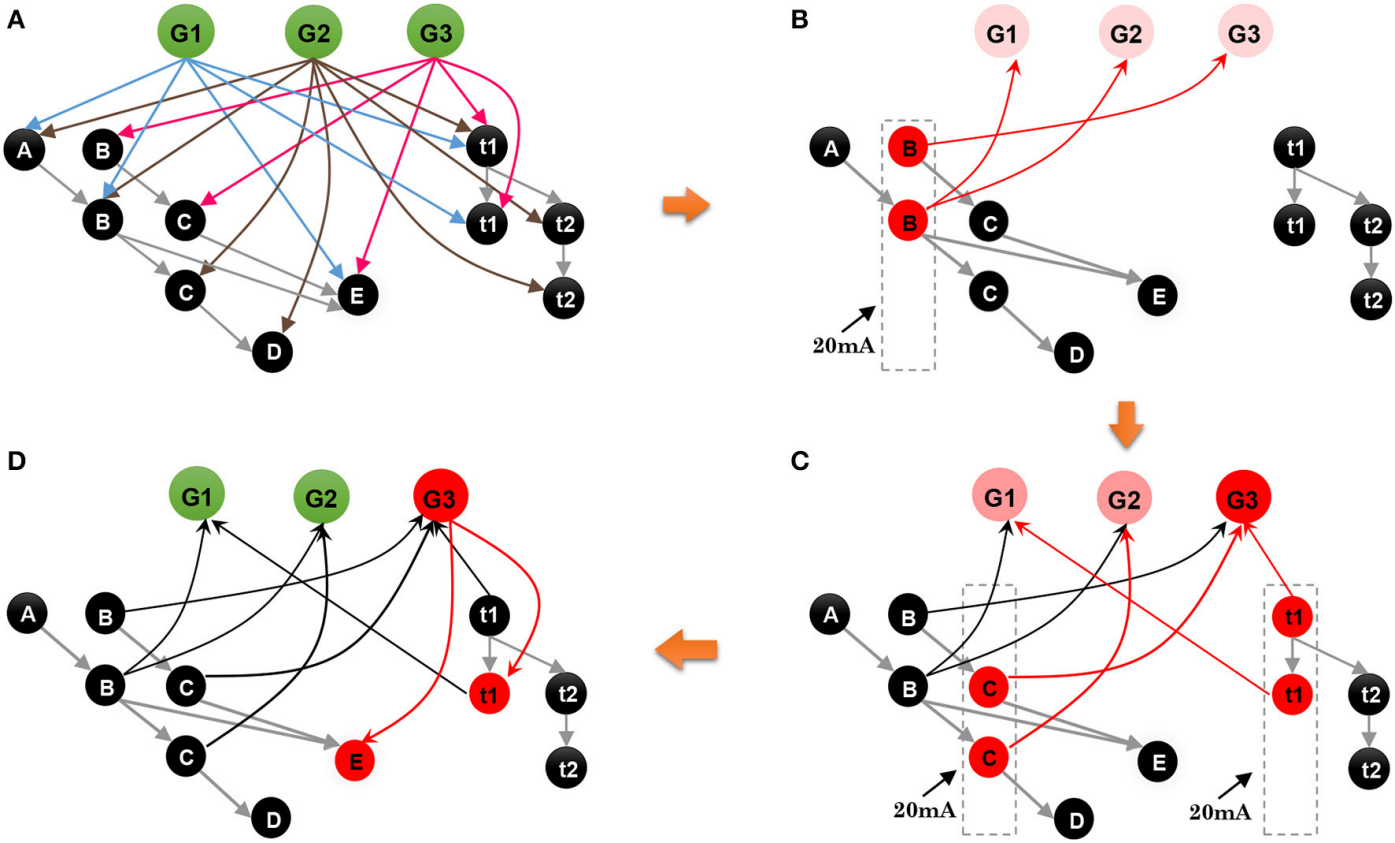
Contextual retrieval: neurons with substantial activities are shown in red. **(A)** State of the model when it has stored three sequences. **(B)** State of the network on the arrival of *B* of the input episode. **(C)** Stimulation of the network by *C* and *t*2. **(D)** Final result of this process.

- **Step 1**. As shown in Figure 9A, where neurons and connections are drawn in a simplified manner, suppose that our model has learned three sequences, *G*_1_ = {*A*, 120 ms, *B*, 120 ms, *E*}, *G*_2_ = {*A*, 120 ms, *B*, 240 ms, *C*, 120 ms, *D*}, and *G*_3_ = {*B*, 120 ms, *C*, 120 ms, *E*}. An episode, {*B*, 120 ms, *C*}, is given to recall the relevant sequence; what does the network do next?
- **Step 2**. As shown in Figure 9B, event *B* of the episode initially stimulates the minicolumn encoding *B* in the spatial subnetwork, and all the neurons in this minicolumn fire and transmit their action potentials along the trained connections marked by red arrows. Then the postsynaptic neurons *G*1, *G*2, and *G*3 are triggered and fire with lower firing rates. Actually, during this step, the postsynaptic neurons *C* and *E* also receive the spikes from their connections, but these neurons cannot be activated since the currents at this moment are not strong enough to trigger their firing or to make them fire regularly and continuously.
- **Step 3**. Event *C* occurs, and the time interval 120 ms stimulates *t*1 in the temporal subnetwork, and, as shown in Figure 9C, neurons encoding *C* and 120 ms in the spatial and temporal subnetworks, respectively, release spikes. Similarly, goal neurons connected to these neurons receive synaptic currents again. Then, the goal neuron *G*3 receives the strongest synaptic currents.
- **Step 4**. With the end of the input episode, neuron *G*3 exhibits the most significant firing rate and wins the competition based on the winner-take-all rule, as shown in Figure 9D. The activity of *G*3 then wakes up the resting neurons *E* as well as their time interval neuron *t*2. In the end, sequence *G*3 is recalled, and the last event *E* and the time interval 120 ms are also remembered.

From this example, we can see that contextual retrieval is an associative process in which the goal cluster and the spatial and temporal subnetworks need to collaborate. Synaptic connections between these clusters play a key role throughout the whole process. A critical issue that needs to be mentioned is that neurons that do not belong to *G*3 may fire since their adjacent neurons release spikes, but their firing rates are very low and their membrane potentials decay over time, and therefore these useless neurons, which can be viewed as noise, will not affect the running process too much.

#### 2.4.3. Temporal Scalable Retrieval

The brain is able to represent time over many scales. For example, a musician can play an instrument at different paces. The neural mechanisms underlying relative time and temporal scaling are not completely understood. However, it has been reported that basal ganglia-cortical-thalamic circuits contribute to time interval encoding (Buhusi and Meck, 2005; Merchant et al., 2013a). In particular, it has been found that striatal populations can encode relative time (Mello et al., 2015) and the activities of the striatum adjust motor behavior to adapt to new intervals. It has been proposed that this process can be explained in terms of a theoretical model known as the striatal beat-frequency model (Matell and Meck, 2004). Although a variety of computational models have been constructed to encode scalable time intervals (Fukai, 1999; Matell and Meck, 2004; Piras and Coull, 2011; Hardy and Buonomano, 2016; Hardy et al., 2018), they are difficult to apply to practical problems.

**Figure 10:**
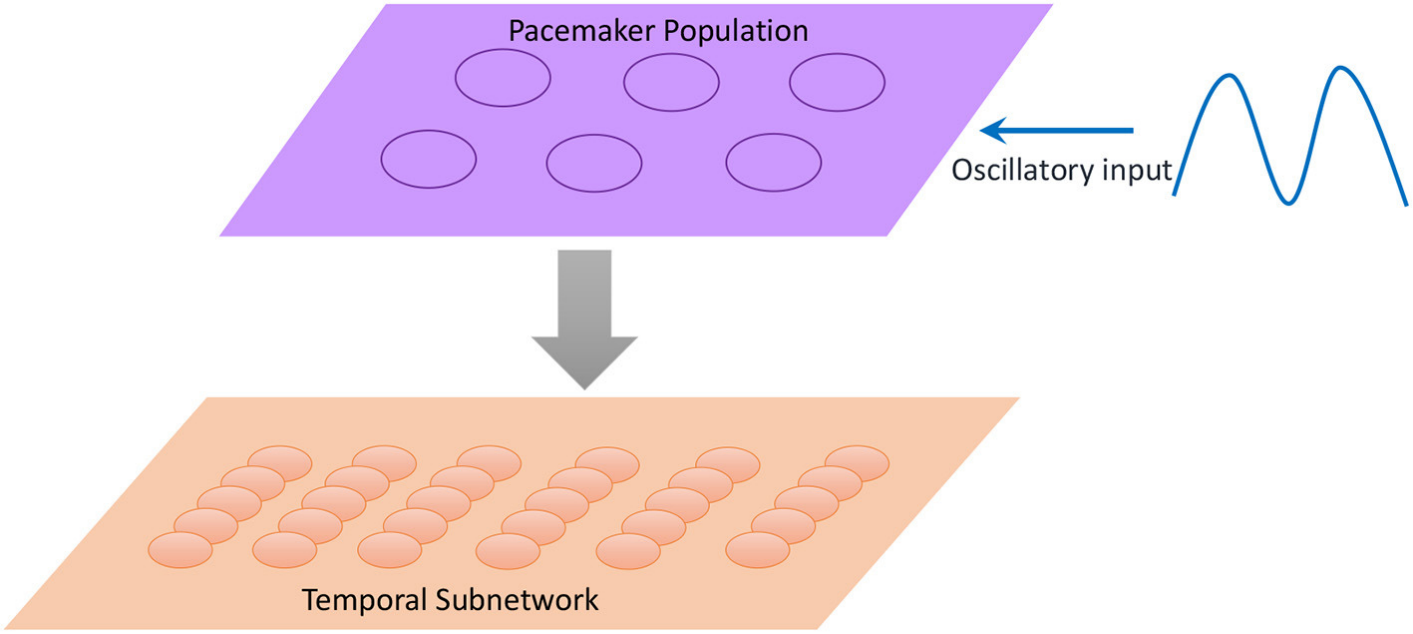
Neurons in the pacemaker cluster project their connections to the temporal subnetwork, and the oscillatory input makes pacemaker neurons exhibit spikes with different frequencies.

Inspired by the neural mechanisms mentioned above, we propose a scalable temporal model to retrieve sequences at different speeds. As illustrated in Figure 10, a neural population called the pacemaker cluster is added to simulate the striatum's function of rescaling time intervals. All neurons in the temporal subnetwork are connected to this population with fixed weights. We adjust the response frequencies of spatial neurons by changing the mean firing rates of pacemaker neurons. This means that the firing rates of pacemaker neurons determine the basic rhythm of the retrieval process. Fast spiking of pacemaker neurons leads to generation of music at a fast pace. In the retrieval process, time intervals encoded by neurons of the temporal subnetwork determine the length of time during which the spatial neurons fire continuously. Rapid activities of temporal neurons caused by the pacemaker population rescale time intervals. To tune the mean firing rate of the pacemaker population, neurons in this cluster are simulated by the integrate-and-fire model, and their membrane potentials obey the equation

(13) $$\begin{array}{l} \tau \frac{dV_{i}}{dt}=-V_{i}+V_{\text{rest}}+V_{\text{osc}} \end{array}$$

where τ is a time constant, *V*_*i*_ is the membrane potential, *V*_rest_ is the resting potential after the neuron has emitted a spike, and *V*_osc_ is an oscillatory input to make the neuron fire at a specific frequency. Here, *V*_osc_ is given by

(14) $$\begin{array}{l} V_{\text{osc}}\left(t\right)=a\cos\left(2\pi ft\right) \end{array}$$

where *a* is the amplitude and *f* is the frequency of oscillation. Hence, the mean firing rate of the pacemaker population can be tuned by *f*.

During the retrieval process, the input current of each temporal neuron is computed as

(15) $$\begin{array}{l} I\left(i\right)=w_{s}I_{\text{same}}+w_{a}I_{\text{adj}}+w_{c}I_{\text{crossing}}+w_{g}I_{\text{goal}}+w_{o}I_{\text{pacemaker}} \end{array}$$

where *w*_*o*_ is the weight of the input from pacemaker neurons and is a constant value, and *I*_pacemaker_ refers to the signals from the pacemaker neurons and is computed as

(16) $$\begin{array}{l} I_{\text{pacemaker}}=\overset{N}{\underset{j=1}{\sum }}\overset{M}{\underset{f=1}{\sum }}\kappa \delta \left(t-t_{j}^{f}\right), \end{array}$$

with κ being the fixed weight of the connection between a temporal neuron and a pacemaker neuron, $t_{j}^{f}$ the spiking time of pacemaker neuron *j* during a 3 ms time window, *N* the number of pacemaker neurons, and *M* the number of spikes emitted by a pacemaker neuron during a time window. The other terms in Equation (15) are defined in the text following Equation (8). Then, the new interval encoded by each temporal neuron can be computed as

(17) $$\begin{array}{l} t_{\text{new}}=c\frac{t_{\text{ori}}}{f} \end{array}$$

where *t*_new_ denotes the new interval of the temporal neurons as tuned by the pacemaker neurons, *c* is a constant coefficient, *t*_ori_ is the length of time that the neuron originally encoded, and *f* is the mean firing rate of this neuron.

## 3. Results

### 3.1. Model Application: Musical Learning

We use musical learning as an example to validate our model. In this paper we mainly consider pure music without lyrics, and the main instrument is the piano; other instruments will be considered in future work.

A musical melody is composed of a series of notes, which have three essential attributes: pitch, duration, and intensity. If we look at Figure 1 and regard the color of a circle there as the pitch of a note and the distance between consecutive circles as the duration of a note (the length of time for which it is played), then a musical melody can be expressed in terms of a spatial pattern and a temporal pattern. Here we ignore the intensities of notes, which will be considered in future work.

It has been found that each neuron in the primary auditory cortex (PAC) has a preferred pitch, and thus the PAC provides a map, which has been called a tonotopic map (Kalat, 2015). However, although the way in which the brain perceives the rhythms of music has been studied for many decades, there remains more controversy than consensus. Numerous neuroscientific experiments have indicated that auditory-motor interactions contribute to rhythm perception (Chen et al., 2006, 2008; Zatorre et al., 2007), but how this mechanism works is still not clear. Therefore, we have to make some assumptions here regarding musical rhythm perception. Inspired by findings mentioned in section 2.2.3, we assume that neurons preferring different time intervals can encode the duration of a note.

**Figure 11:**
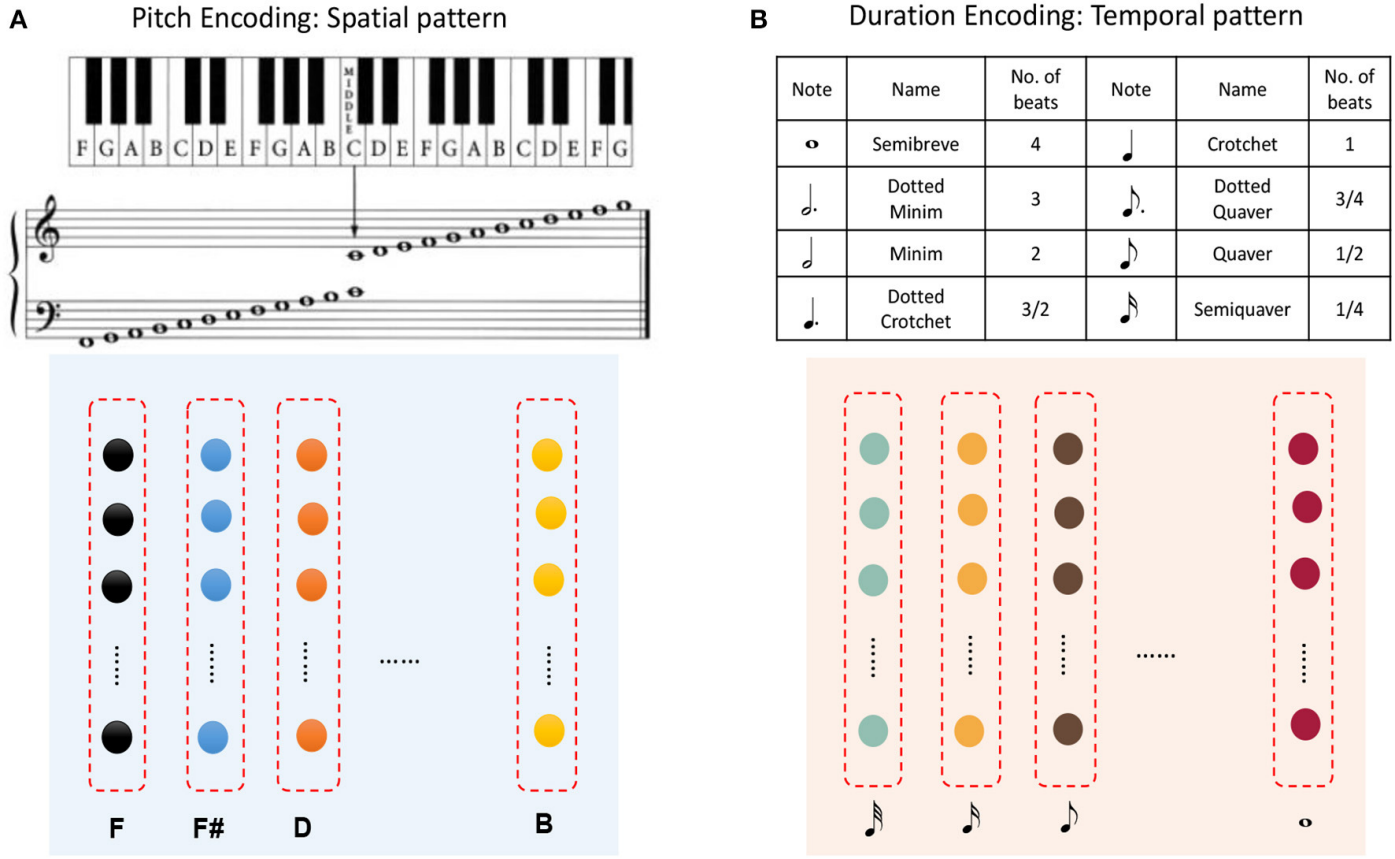
Application to musical learning. **(A)** Encoding of the pitch of a note using a spatial minicolumn. **(B)** Representation of the duration of a note as the preference of a temporal minicolumn.

Figure 11A shows that pitches can be encoded by minicolumns of the spatial subnetwork. Each minicolumn has its preferred pitch. The spatial subnetwork has 88 minicolumns to encode pitches corresponding to the 88 keys on a piano keyboard. Equation (6) is used to compute the injected currents of neurons, and the input value of this equation is the pitch frequency. Similarly, the durations of notes can be encoded by minicolumns of the temporal subnetwork. It is important to note that we cannot define the absolute number of milliseconds for which a note lasts, which depends on how fast a performer is playing the instrument. As is shown in Figure 11B, the time perceived by each temporal minicolumn is related to the number of beats. As a rule of thumb, one crochet generally lasts from a few hundred milliseconds to a few seconds.

### 3.2. Experiments

The dataset used in this paper is derived from a classical piano database (Krueger, 2018). We collect 331 MIDI files of classical piano melodies to validate our model. MIDI, the Musical Instrument Digital Interface, is a standard protocol that connects digital musical devices and computers. MIDI files contain lists of instructions for tracks, notes, meters, and instruments, together with other data, which can represent complete musical information for users^1^.

**Figure 12:**
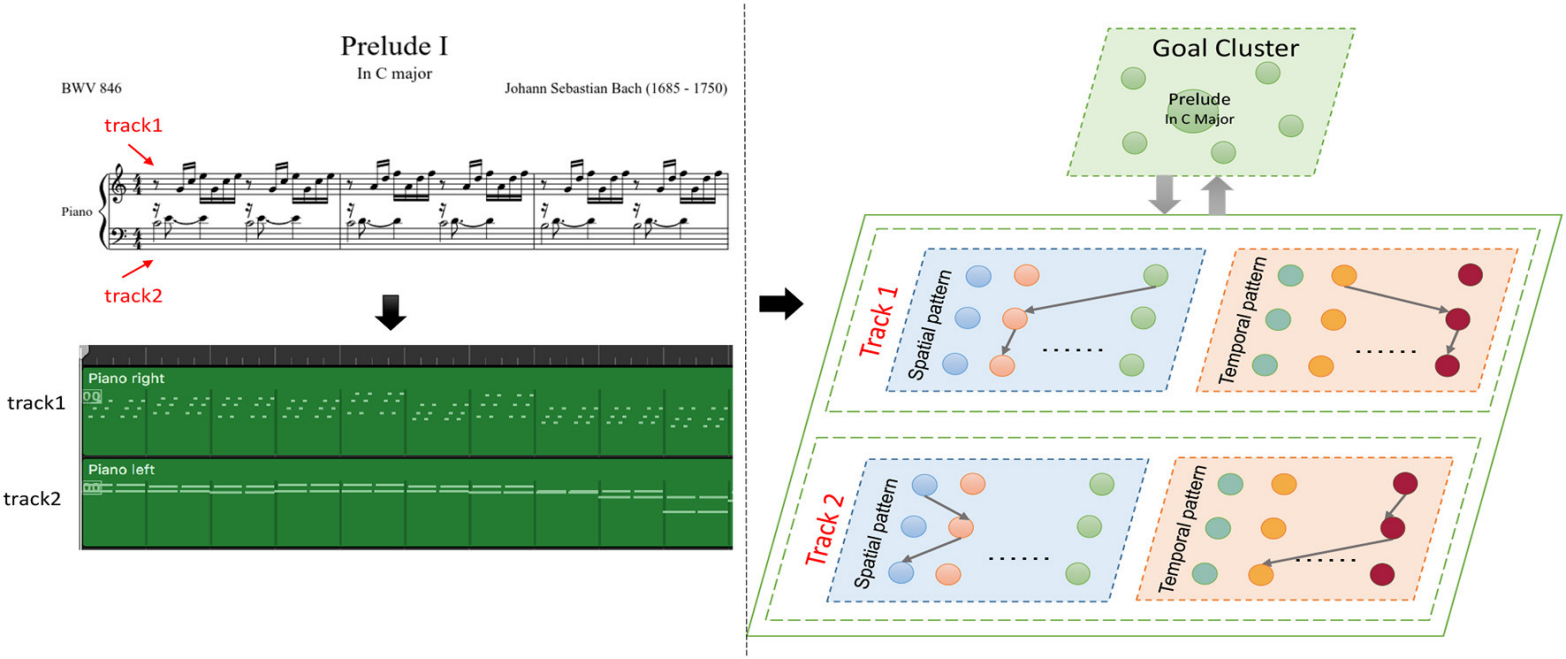
Episode of Bach's BWV 846: the green graph describes the tracks of this work in MIDI files, and the model creates spatial and temporal subnetworks for each track.

As shown in Figure 12, a musical melody always has multiple parts represented as tracks in the MIDI file. We create spatial and temporal subnetworks for each track, and the goal cluster stores the names of the melodies. These subnetworks work together during the whole process. For simplicity, we take the first track as an example to describe the experiments.

#### 3.2.1. Encoding

A MIDI file defines 128 pitches, which are represented by the digits 0–127. However, our spatial subnetwork is composed of 88 minicolumns to encode the standard pitches corresponding to the piano keys. We use the pitch index defined by the MIDI standard, rather than the pitch frequency, as a spatial neuronal preference. The encoding process of the spatial subnetwork thereby becomes straightforward and precise.

**Figure 13:**
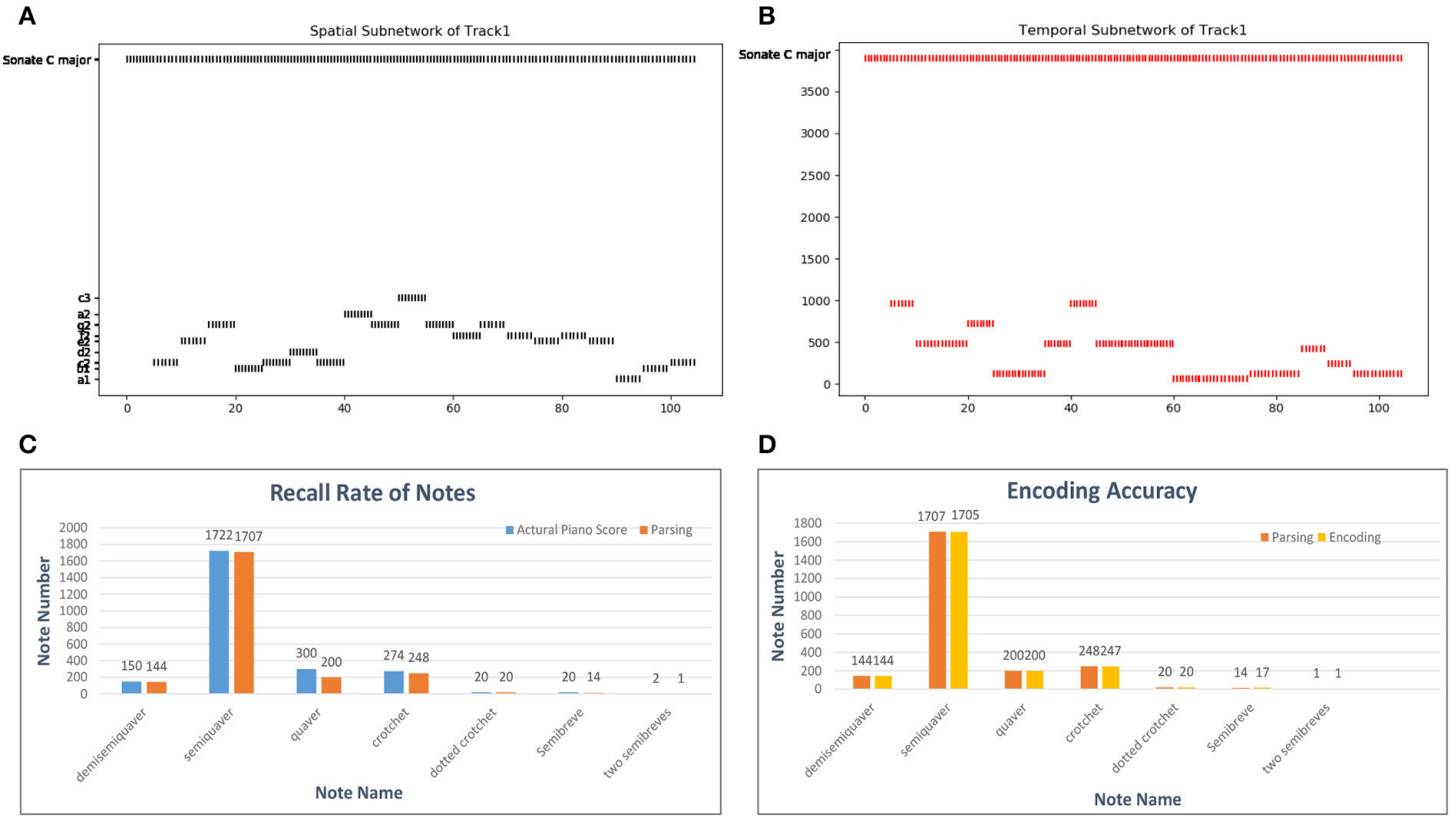
Results of the encoding process. **(A,B)** Activities of neurons in the spatial and temporal subnetworks, respectively, when we input Mozart's KV 545_1. The horizontal axis represents the simulation time and the vertical axis the information encoded by neurons in these two subnetworks. **(C)** Parsing accuracy of the MIDI files. The labels on the horizontal axis are the names of works in our dataset; we show only some of these labels because of space limitations. **(D)** Encoding accuracy of our model in general.

A MIDI file also defines the number of ticks (usually 480) for which a crotchet lasts as the time reference. According to this basic crotchet unit, we create 64 temporal minicolumns that can encode note durations from a demisemiquaver to two semibreves. Here, we use a classical piano work, Mozart's Sonata No. 16 in C Major, KV 545, to estimate the encoding ability of temporal neurons. The encoding process is shown in Figures 13A,B: as the simulated time passes, the neurons of the spatial and temporal subnetworks fire when they receive the external information. The neuron encoding the name of this melody in the goal cluster is excited throughout the process. For simplicity, this figure shows only 20 notes. In addition, since there are some errors (tenuto, broken chords, etc.) during the parsing process of the MIDI file, we first compute the recall rate of the parsing process. According to the piano score, this work includes 2, 488 notes; however, 2, 502 notes are resolved out by parsing the related MIDI file. The reason this discrepancy occurs is that chords are divided into individual notes during the parsing process. Because the parsing algorithm is not precise and musical pieces recorded in MIDI files are played by a human, this process cannot be completely accurate. The recall rates of notes of different durations are shown in Figure 13C, and the total recall rate is 93.81%. Figure 13D shows the encoding accuracy of note duration based on the recalled notes; the average encoding accuracy for this work is 99.87%.

#### 3.2.2. Storage

**Figure 14:**
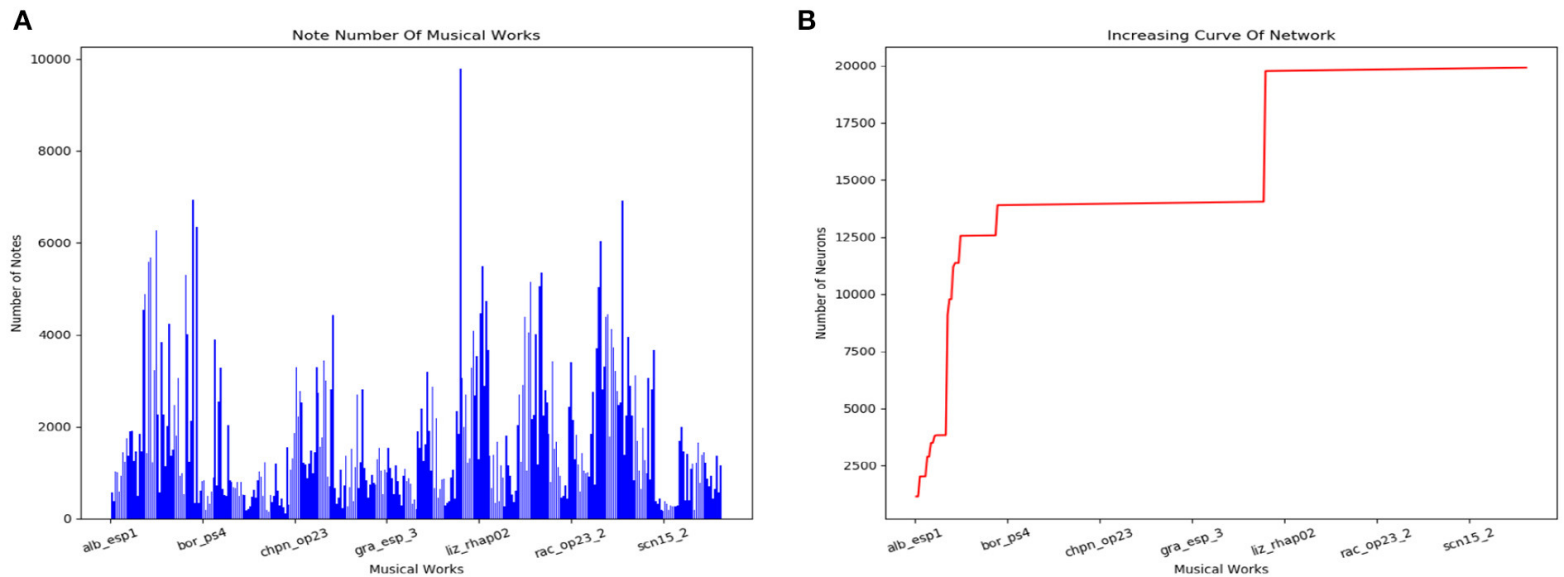
Results of the storage process. **(A)** Number of notes in each classical work in our dataset. **(B)** Increasing curve of the scale of our model during the learning process.

The scale of the model changes during the storage process. Figure 14A counts the number of notes of every musical work in the dataset. The increasing curve of the number of neurons during the learning process is shown in Figure 14B. We can conclude that the size of the network does not grow indefinitely with the amount of training data. The total number of neurons depends on the longest musical melody. Based on our dataset, the model finally consists of about 20,000 neurons.

#### 3.2.3. Retrieval

**Figure 15:**
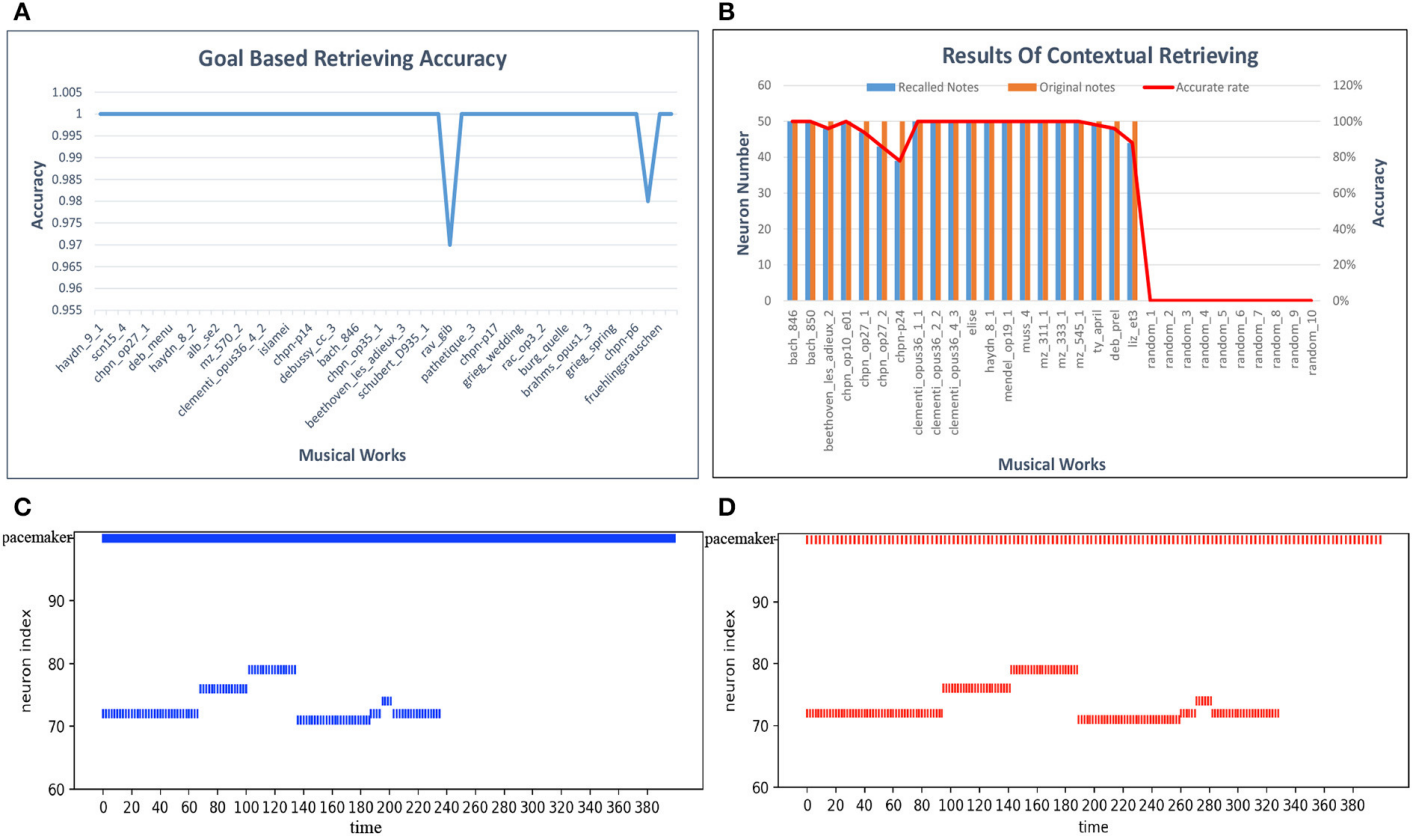
Results of retrieval. **(A)** Accuracy of goal-based retrieval. **(B)** Contextual retrieval based on our test set. **(C)** A fast firing rate leads to retrieval at a fast speed. **(D)** Adjustment of the retrieval process to a low pace.

- **Goal-based retrieval**
  For the goal-based retrieval process, we take 50 musical pieces selected randomly from the dataset. The model shows the first 100 notes, including pitch and duration information, according to the input titles of the musical pieces. We first calculate the retrieval accuracy of each musical work, and the results are plotted in Figure 15A. The retrieval accuracy for most of the musical works is 100% because synapses between the goal cluster and the spatial and temporal subnetworks play an important role. Based on these results, the average accuracy of the goal-based retrieval is 99.9%.
- **Contextual retrieval**
  For contextual retrieval, the test set is a collection of short musical episodes played by us and recorded in MIDI files. There are 30 MIDI files in the test set: 20 pieces are derived from the artistic works in our dataset, and the others come from Chinese or pop music. The length of each test piece is not fixed. The model shows the rest of the 50 notes and the name information after input of the test episodes. Both the pitch and the duration of a note must be recalled correctly, or else we consider that the note has failed to be recalled. Figure 15B shows details of this process, with blue bars indicating the notes that should be recalled and red bars the notes retrieved by our model; random_1 to random_10 correspond to pieces played randomly by one of us. According to these results, the notes and goal information can generally be recalled accurately. However, compared with other works, the retrieval accuracy of pieces by Chopin is relatively low, since they tend to be full of variations (tercets or various slurs). Errors may occur during the course of the retrieval process. Based on these experiments, the average accuracy of contextual retrieval is about 97%.
- **Temporal scalable retrieval**
  We again use Mozart's Sonata No. 16 in C Major, KV 545, to test this process. We modulate the firing rate of the pacemaker population, and the speed of retrieval is then changed correspondingly. Figures 15C,D show seven retrieved notes (the first phrase) of the first track of this piano piece when the parameter *f* of the oscillatory input is set to 8.8 and 3, respectively, in Equation (14). The ordinate represents the spatial neuron index, which corresponds to the pitch index defined in the MIDI format. The horizontal axis represents the simulation time (in milliseconds). These two graphs are simplified to show only the mean firing rate of the pacemaker population. Compared with Figure 15C, the retrieval of notes in Figure 15D takes more time. Correspondingly, the intervals between the successive pitches are prolonged.

## 4. Discussion

This paper introduces a spiking neural model of sequential memory based on brain mechanisms. Musical learning is used as a typical application to evaluate the network. Four neural clusters, namely, a goal cluster, a pacemaker cluster, and spatial and temporal subnetworks, are involved in encoding, storage, and retrieval sequences. Minicolumns with different preferences encode the contents and time lengths of sequences. The connection architecture, which takes into account not only ordered information but also sequential context, can store a large number of sequences. An STDP learning rule is adopted to update connection weights during the memorization process. Experiments show that the model can store a large number of musical melodies. Because of the associative property of the network, both goal-based and contextual retrieval give highly accurate results. The melody can be retrieved at different speeds by tuning the frequency of the pacemaker population, and this process makes the model behave similarly to human memory.

In theory, the model can store a very large number of sequences. The scale of the network varies with the number of input sequences. If there are *n* elements and *m* is the length of the longest sequence, then the model can store *n*^*m*^ different sequences. However, if we take musical learning as an example, we can see that it is impossible for a musical melody to consist of the full arrangement of notes. The capacity of the model will not reach saturation, but will be limited by *m*. This issue will be considered in our future work.

Sequential memory is a fundamental cognitive process of the brain. It involves many interesting and important aspects, some of which will be examined in our future work.

- We plan to construct a hierarchically structured model that is able to process bottom-up sequential information. Neural assemblies distributed at high levels can represent more advanced information. For example, in a music learning problem, notes, meters, phrases, sections, and even movements can be learned using such a hierarchical model.
- Time perception is a key issue in sequential memory. We believe that the mechanism of time perception is very complex and needs to be studied more deeply in future work. For example, it has been found that neural circuits with conduction delays are able to encode time information. This mechanism can cooperate with the temporal subnetwork and basal ganglia to allow a more precise perception of time. We aim to improve our model on the basis of these findings. Additionally, real-time simulation needs to be considered, although this is a challenging problem.
- In its application to music learning, the model will focus on representation of chords and pauses. An essential component of this task is finding out how to represent and learn chords. Pauses are also present throughout musical pieces, and these need to be taken into account in future development of the model. Another essential topic to be dealt with is that of musical generation.
- Forgetting mechanisms also need to be incorporated into the model. At present, the model is able to allocate new neurons to store further information; how it can be modified to delete or alter neurons will be considered in future work.

## Data Availability Statement

Publicly available datasets were analyzed in this study. This data can be found here: http://www.piano-midi.de/.

## Author Contributions

QL and YZ designed the study and performed the experiments. QL, YZ, and BX developed the algorithm and performed the analysis of the results. QL and YZ wrote the manuscript.

## Conflict of Interest

The authors declare that the research was conducted in the absence of any commercial or financial relationships that could be construed as a potential conflict of interest.

## Footnotes

^1^Further details can be found at https://www.midi.org/.
